# On the Proportion of Cases of Small-Pox after Vaccination, &c. &c.

**Published:** 1827-05

**Authors:** Edward Morton

**Affiliations:** Physician to the Western Dispensary, and to the Royal Metropolitan Infirmary for Children, &c.


*On the Proportion of Cases of Small-Pox after Vaccination, &c. &c.* By EDWARD MORTON, M.B. L.M. Physician to the WESTERN DISPENSARY, and to the ROYAL METROPOLITAN INFIRMARY FOR CHILDREN, &c.

THAT cases of small-pox frequently happen after vaccination, is a fact that is now universally admitted among the profession; but the proportion in which these occur has not, I believe, as yet been correctly ascertained. As this is a question which can alone be decided by careful observation, I beg to submit to your readers the result of my experience on the subject during the last year; premising that it is derived from notes made at the time from cases passing under my eye, or from my own personal inquiries.

From the 1st of April, 1826, to the same date in the present year, there have been admitted, under my care, at the Royal Infirmary, 810 children, of or under twelve years of age. Of these, 126 had the natural small-pox; 22 the inoculated small-pox; 261 remained unprotected by either; and 401 have been vaccinated. The latter will alone engage our attention upon the present occasion.

Out of the above-mentioned 401 children who have been subjected to vaccination, seventeen have had small-pox afterwards. Of these, ten have merely been reported to me as such upon their admission; the remaining seven have been seen and attended by myself. Allowing, therefore, all the cases above referred to actually to have occurred, (and I have no reason whatever to doubt the fact,) it will be seen that the instances of small-pox that have occurred subsequently to vaccination, out of the above number of children, have been only four and a fraction in every hundred. If the accuracy of the reported cases be questioned, then there will have been less than two in the hundred. In either case, the proportion is so small as not to invalidate, in the slightest degree, the general efficacy of vaccination.

Of the foregoing cases, those which did not fall under my own observation were uniformly represented as having been extremely mild, and quick in passing through their different stages, the children seldom being at any period confined to their beds; and those that I myself superintended were of exactly the same description. One of the patients, six months old, had only two pocks; while her elder sister, from whom she received the infection, who had not been vaccinated, died of confluent small-pox. If therefore vaccination, even when successfully practised, do not invariably preclude, at any future period, the occurrence of small-pox, (for I must observe, that I have seen several cases, some of which will be mentioned hereafter, where it has happened in children whose arms have exhibited the scars supposed to indicate a perfect and correct performance of the operation,) yet it is unquestionable that it is capable of preventing all the dangerous symptoms of that dreadful malady; that the subsequent disease is uniformly a mild one, and has in no instance, within the knowledge of my professional friends or myself, ever proved fatal.

The following cases, which have come to my knowledge within the last twelve months, appear to support the opinion of Dr. Gregory and others, that small-pox after vaccination happens only in particular families; and that there exists in these a peculiarity of constitution which predisposes them, in an unusual degree, to be attacked by it. They also seem to render it probable that parents, who have themselves suffered from the ravages of small-pox, may be able to impart to their offspring, in consequence, a predisposition to it.

I.—Sarah Lumley, ætatis five years, was admitted June 6th, 1826, for modified small-pox: had 120 pocks, perfectly distinct, which did not observe the regular stages. She soon recovered perfectly. This child, when two years of age, was vaccinated at Rowland Hill's Chapel, and had a card given to show that the operation had been successfully performed.

Her younger brother, who had not been vaccinated, soon afterwards became affected with confluent small-pox, (apparently in consequence of infection received from her,) and was attended by myself.

The mother of these children informed me, that some years ago she had one of her children inoculated with the small-pox; that two others took the infection from her, and died;* that she herself, in her youth, underwent small-pox, and suffered severely from it; and that her husband also passed through it, and is much pitted in consequence.

II.—From the mother of a patient of the name of King, who had small-pox after vaccination, I learned that she herself had under-

* "Died." So here the mother, in attempting to protect one child by inoculation, was actually the means of destroying two, who possibly might never have casually taken the disease.

gone small-pox in a severe manner; that her husband was also much pitted with it; and that three of her children, who had been previously vaccinated, passed through small-pox afterwards, but soon recovered.

III.—William Luke, ætatis five years, was admitted May 23d, 1826, for modified small-pox, (having been previously vaccinated at the Small-Pox Hospital,) and was soon well. Has three distinct perfect scars in the left arm, and two in the right.

The sister of the above was also vaccinated at the Small-Pox Hospital when an infant, and now exhibits four small, indented, circular scars in the left arm. About three years ago, she underwent small-pox also, and had a great many pustules, (the mother counted thirty-six upon one arm,) but soon recovered.

The father of these children has never had the disease; but the mother had it severely, and is thickly and deeply pitted.

IV.—Mary Malony, ætatis one year and six months, was admitted December 15th, 1826, for modified small-pox, and was speedily discharged cured. This child was vaccinated about a year previously. The operation was performed in both arms, but only one pock arose in the left, which now exhibits a proper circular scar.

John and Ellen, the brother and sister of the above, were also reported to me by the mother as having had small-pox after vaccination; which she described as resembling the eruption which I witnessed, during its progress, in Mary.

V.—Jonathan and Richard Cooper, two brothers, admitted in March 1827, are also among the numbers of those reported to have had small-pox after vaccination, but in which the disease presented nothing remarkable.

15, *Eaton-street, Grosvenor-place;*

*April 7th,* 1827.

